# Evaluation After Cochlear Implant Surgery: Correlation of Clinical Outcome and Imaging Findings using Flat-detector CT

**DOI:** 10.1007/s00062-020-00922-1

**Published:** 2020-06-15

**Authors:** Annika Stock, Victoria Bozzato, Stephan P. Kloska, Alessandro Bozzato, Ulrich Hoppe, Joachim Hornung, Arnd Dörfler, Tobias Struffert

**Affiliations:** 1 Department of Neuroradiology, University Hospital Erlangen-Nuremberg, Erlangen, Germany; 2 Department of Neuroradiology, University Hospital Würzburg, Josef-Schneider-Straße 11, 97080 Würzburg, Germany; 3 Department of Otorhinolaryngology, Head and Neck Surgery, University Hospital Saarland Medical School, Homburg, Germany; 4 Department of Radiology, Hospital Fuerth, Fürth, Germany; 5 Department of Otorhinolaryngology, Head & Neck Surgery, University of Erlangen-Nuremberg, Erlangen, Germany; 6 Department of Neuroradiology, University Hospital Gießen, Gießen, Germany

**Keywords:** Cochlear implantation, Deafness, Rehabilitation, Dyna-CT, Misplaced cochlear implant electrode array

## Abstract

**Purpose:**

Assessment of the cochlear implant (CI) electrode array position using flat-detector computed tomography (FDCT) to test dependence of postoperative outcome on intracochlear electrode position.

**Methods:**

A total of 102 patients implanted with 107 CIs underwent FDCT. Electrode position was rated as 1) scala tympani, 2) scala vestibuli, 3) scalar dislocation and 4) no deconvolution. Two independent neuroradiologists rated all image data sets twice and the scalar position was verified by a third neuroradiologist. Presurgical and postsurgical speech audiometry by the Freiburg monosyllabic test was used to evaluate auditory outcome after 6 months of speech rehabilitation.

**Results:**

Electrode array position was assessed by FDCT in 107 CIs. Of the electrodes 60 were detected in the scala tympani, 21 in the scala vestibuli, 24 electrode arrays showed scalar dislocation and 2 electrodes were not placed in an intracochlear position. There was no significant difference in rehabilitation outcomes between scala tympani and scala vestibuli inserted patients. Rehabilitation was also possible in patients with dislocated electrodes.

**Conclusion:**

The use of FDCT is a reliable diagnostic method to determine the position of the electrode array. In our study cohort, the electrode position had no significant impact on postoperative outcome except for non-deconvoluted electrode arrays.

## Introduction

Cochlear implants (CIs) enable the restoration of hearing in patients with severe hearing loss or deafness. Nevertheless, patients’ hearing improvement after surgery and rehabilitation may vary. There is ongoing discussion on how the scalar positioning of CI electrodes affects the quality of postoperative auditory rehabilitation. Due to the anatomical conditions and the surgical technique, electrode array insertion into the scala tympani (ST) has become standard [1, 2]. ST insertion has been described as minimally traumatic [3]. This is necessary to protect the fine bony structures such as the osseous spiral lamina and to prevent neuronal degeneration. Earlier clinical studies have reported optimal auditory outcome with ST insertions, and lower outcome scores have been associated with a greater number of electrode contacts in the scala vestibuli (SV) [4, 5]. Since ST implantation is not possible in every case, for example in cases of cochlear ossification or malformation, some authors reported similar good rehabilitation results with SV insertions [6, 7]. Furthermore, one pediatric study showed that the speech performance did not correlate to the intracochlear positioning (ST versus SV) [8].

In the clinical routine, the exact electrode array position is relevant, especially in cases of implant malfunction; however, the discrimination of SV and ST by conventional multi-slice computed tomography (MSCT) is complicated by the limited spatial resolution and metal artifacts that blur the surroundings of the implant. Therefore, MSCT does not allow exact assessment of electrode array position in every case [9]. Due to its superior spatial resolution, flat-detector computed tomography (FDCT) provides improved imaging quality of the fine bony structures of the temporal bone [10, 11]. In addition, metal artifacts have less impact on cochlear assessment in FDCT compared to MSCT [12]. It has been shown that the higher image quality allows an improved assessment of the electrode array position, that single electrode contacts are visible and that the radiation dose is essentially lower in FDCT compared to MSCT [12, 13].

The aim of this retrospective study was 1) to analyze to what extent the higher spatial resolution of the FDCT enables precise determination of the electrode array position and 2) to correlate scalar positioning with the patient’s auditory outcome.

## Material and Method

### Patients

Patients implanted with the Nucleus Contour Advance device (Cochlear Ltd, Lane Cove, NSW, Australia) were included regardless of age, reason and date/period of deafness/hearing loss. All patients, as per institutional standard, underwent FDCT postoperatively and were only included with complete speech audiometry using the Freiburg monosyllabic test (FMT) in quiet presurgery and 6 months postsurgery. Monaural hearing ability was tested preoperatively with a hearing aid (in cases of residual hearing) and postoperatively with CI. The improvement of hearing ability after a rehabilitation period of 6 months, here called outcome, was determined by the difference in preoperative and postoperative monosyllabic recognition scores at 65 dB (%) in quiet.

### Flat-Detector CT

**Fig. 1:**
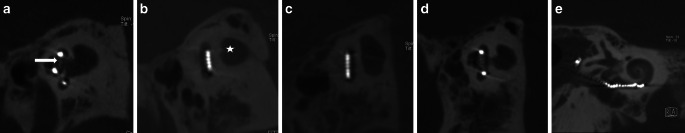
Examples for electrode array positions. **a** The arrow marks the osseous spiral lamina. **b** ST position, the electrode array is adjacent to the internal acoustic meatus (*white star*) in coronal reconstruction. **c** SV position in coronal reconstruction. **d** Scalar dislocation of the electrode array in coronal reconstruction, one electrode contact is located in SV and one in ST. **e** Electrode array at the cochlear base (sagittal reconstruction) rated as no deconvolution

The FDCT was performed on a biplane flat-detector angiography system (Axiom Artis dBA, Siemens Healthcare AG, Forchheim, Germany). Reconstructions and postprocessing of data sets were performed using the standard software of the manufacturer (Syngo InSpace, Syngo 3D; Siemens Healthcare GmbH) on a standard workstation. Multiplanar reconstructions (MPR) were processed in axial, sagittal and coronal planes with 1 mm reconstruction slice thickness and spacing. The MPR images were stored anonymously on a workstation. Windowing was left to the preference of the reviewer to obtain the best image impression. Electrode array position was rated as: 1) ST, 2) SV, 3) scalar dislocation and 4) no deconvolution. The osseous spiral lamina, separating ST and SV, and the internal acoustic meatus were reference points for assessing implant localization. The coronal plane is appropriate for this purpose. If the electrode array is located on the side of the spiral osseous lamina adjacent to the internal acoustic meatus, it can be assumed that the implant is positioned in the ST. If the electrode array crosses the spiral osseous lamina and single electrode contacts are found in ST and SV, the implant is dislocated (examples for each position are given in Fig. 1).

### Statistical Methods

Two independent neuroradiologists (R1 and R2) assessed all image data sets twice in an interval of 2 months (assessments named: R1/1, R1/2; R2/1, R2/2). Furthermore, a third reviewer (T. S.), most experienced in FDCT imaging of the temporal bone, assessed all data sets, especially to achieve consensus in cases of divergent results between R1 and R2.

Using Cohens-Kappa test, intrarater reliability and interrater reliability were tested for stability and reliability of the imaging method. Results were rated as: slight agreement (κ = 0–0.2), fair agreement (κ = 0.21–0.4), moderate agreement (κ = 0.41–0.6), substantial agreement (κ = 0.61–0.8) and almost perfect agreement (κ = 0.81–1.0) [14].

The hearing ability of each patient was evaluated presurgery and 6 months after by speech audiometry (FMT) to determine the auditory outcome. Dependence between outcome and electrode positioning was tested with the χ^2^-test, a significance level of 0.05 was chosen.

## Results

Test results and image datasets were available in 102 patients, 5 patients received a CI bilaterally (number of inserted CIs *n* = 107), 58 patients were male, 1 received a CI bilaterally and 44 were female, 4 received a CI bilaterally. Patients age ranged from 17 to 86 years (median 56.0 years). Our cohort had a wide range of causes of deafness (Table 1). Highest prevalence in our study was the progressive sensorineural hearing loss (progressive SNHL 62.6%).

**Table 1:** Causes of hearing loss/deafness (*n* = number of CIs)

Cause	Example	*n*	%
Progressive SNHL		67	62.6
Postsurgery	After stapes operation	8	7.5
Infectious	Meningitis	8	7.5
Traumatic	Traumatic brain injury	3	2.8
Drug-induced	Aminoglycosamide	2	1.9
Sudden hearing loss	Acoustic trauma	3	2.8
Hereditary hearing loss		10	9.3
Others	Neurofibromatosis type I	6	5.6

*SNHL* sensorineural hearing loss

All patients underwent FDCT postoperatively to evaluate electrode array position. Assessment of electrode array position was successful for each CI and 60 (56.1%) electrode arrays were positioned in the ST. Electrode array insertion into the SV was detected in 21 (19.6%) and 24 (22.4%) electrode arrays showed scalar dislocation (dislocation from ST to SV). Two electrode arrays were not placed in the cochlea but results were included in this evaluation. One electrode array ended at the cochlear base, this patient showed a cochlear malformation, and the other electrode array was completely distanced to the cochlea. Both electrode arrays were rated as non-deconvoluted. Intrarater and interrater reliability of electrode positioning of each assessment showed an almost perfect agreement (results in Table 2). In 16 cases, a consensus between R1 and R2 was necessary. In 10 of these cases, T. S. rated the electrode array as dislocated. Furthermore, in six cases interrater and intrarater results (R1/R2) were different. In five of these six cases, T. S. assessed scalar dislocation. Only two out of six cases showed metal artifacts that partially limited the assessment. An overview of the single ratings is given in Table 3.

**Table 2:** Intrarater and interrater reliability results (κ)

**Intrarater reliability**	**κ**
R1/1–R1/2	0.81
R2/1–R2/2	0.95
**Interrater reliability**	**κ**
R1/1–R2/1	0.85
R1/2–R2/2	0.87
T. S.–R1/1	0.92
T. S.–R1/2	0.89
T. S.–R2/1	0.87
T. S.–R2/2	0.86

**Table 3:** Results of the evaluation of intracochlear electrode array positioning

Electrode position (*n* = 107)	R1/1	R1/2	R2/1	R2/2	T. S.
Scala tympani	62 (57.9%)	60 (56.1%)	64 (59.8%)	63 (58.9%)	60 (56.1%)
Scala vestibuli	22 (20.6%)	22 (20.6%)	23 (21.5%)	23 (21.5%)	21 (19.6%)
Scalar dislocation	21 (19.6%)	23 (21.5%)	18 (16.8%)	19 (17.8%)	24 (22.4%)
No deconvolution	2 (1.9%)	2 (1.9%)	2 (1.9%)	2 (1.9%)	2 (1.9%)

Five patients showed cochlear dysplasia (2 ST, 2 SV, 1 scalar dislocation). The outcome ranged from 0% to 65% at 65 dB in quiet (Table 4). One patient showed a dysplastic lateral semicircular canal and achieved an outcome of 45% at 65 dB in quiet.

**Table 4:** Patient outcomes with inner ear dysplasia

Patient nr.		Electrode array position	Outcome at 65 dB (%) in quiet
9	Dysplastic cochlea	Scala tympani	0
12	Dysplastic cochlea	Scala vestibuli	65
18	Dysplastic lateral semicircular canal	Scala tympani	45
26	Dysplastic cochlea	Scala vestibuli	0
51	Dysplastic cochlea	Scala tympani	0
98	Dysplastic cochlea	Scalar dislocation	30

**Fig. 2:**
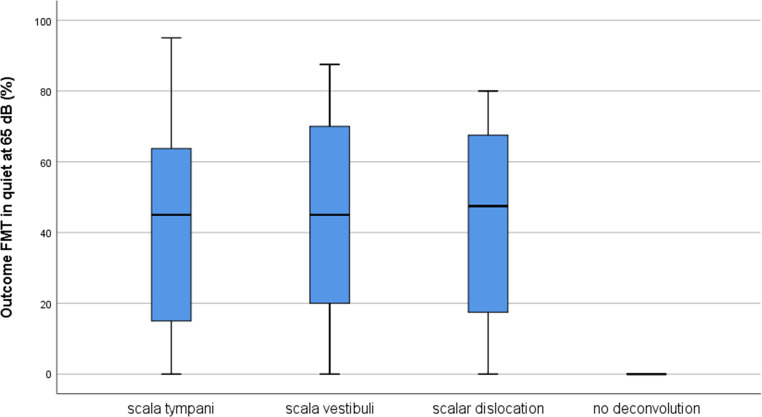
Outcome after 6 months of rehabilitation. Postoperative results of monosyllabic recognition as outcome FMT in quiet at 65 dB (%) for scala tympani (median 45% at 65 dB in quiet, range 0–95; IQR 15–64.4), scala vestibuli (median 45% at 65 dB in quiet, range 0–87.5; IQR 20–70), scalar dislocated (median 47.5% at 65 dB in quiet, range 0–80; IQR 16.3–68.8) and non-deconvoluted electrode arrays after 6 months of rehabilitation

No patient showed a decrease in monosyllabic recognition postoperatively. The median outcome for ST insertion was 45% (range 0–95%) at 65 dB in quiet, for SV insertion 45% (range 0–87.5%) at 65 dB in quiet and for scalar dislocation 47.5% (range 0–80%) at 65 dB in quiet (results in Table 5, illustrated in Fig. 2). Two patients with failed electrode deconvolution had no improvement at all. The outcome was not dependent on the electrode array position. There was no significant difference in outcome between ST, SV or dislocated electrode arrays (R1/1 *p* = 0.57; R1/2 *p* = 0.66; R2/1 *p* = 0.57; R2/2 *p* = 0.72; T. S. *p* = 0.51, χ^2^-test), except for failed deconvolution.

**Table 5:** Overview of monosyllabic comprehension preoperatively (FMT0) and after 6 months of rehabilitation (FMT6)

		Minimum (%)	Maximum (%)	Median
Scala tympani	*n* = 60			
	FMT0	0	45	0
	FMT6	0	95	45
	Outcome^a^	0	95	45
Scala vestibuli	*n* = 21			
	FMT0	0	15	0
	FMT6	0	87.5	52.5
	Outcome^a^	0	87.5	45
Scalar dislocation	*n* = 24			
	FMT0	0	35	0
	FMT6	0	90	61
	Outcome^a^	0	80	47.5

^a^The difference of FMT6 and FMT0 was rated as auditory outcome

## Discussion

To preserve residual hearing through atraumatic electrode array placement and because of the easy access, the ST insertion has become the standard in CI surgery, but some studies reported equal results for SV insertion as well [6–8]. Therefore, the aim of this study was to investigate the impact of CI positioning on the auditory outcome. To our knowledge, this is the largest cohort of CI implanted patients in whom the position of the electrode array was assessed by FDCT.

We used FDCT imaging, which is routinely performed on CI patients in our department, to determine the exact electrode positioning after cochlear implant surgery. The use of FDCT allows exact scalar discrimination in comparison to MSCT [12, 13]. Additionally, high interrater and intrarater reliability in our study indicate a good reliability of this imaging technique, which is necessary in postoperative imaging to validate the intrascalar position of the electrode array; however, our results indicate that it can be challenging to detect scalar dislocation despite the higher resolution of FDCT.

Only patients with a Nucleus Contour Advance device were included. The insertion depth, which is a significant factor [15], the surgeon’s experience and the surgical access were not considered in our evaluation. We observed fewer ST insertions in comparison to the results of Aschendorff et al. (56.1% versus 72.7%) [4] and SV insertions, which are mostly unintentional, occurred in our cohort in 19.6%. Nordfalk et al. achieved similar results (23.1%; 3/13) in an evaluation with different CI models [16]. The number of dislocated electrode arrays in this evaluation (22.4%) was similar in comparison to previous studies [4].

Our results showed no significant difference in auditory outcome between the different electrode localizations (except for non-deconvoluted electrode arrays). These results disagree with previous studies reporting poorer rehabilitation results in case of failed insertions. Aschendorff et al. postulated poor results for SV insertions as well as for scalar dislocation in comparison to ST insertions [4]; however, Adunka et al. inserted electrode arrays into specimens of the human temporal bone and assessed SV insertion as relatively atraumatic, since only the basal structures of the cochlea were damaged [17]. This observation might explain the good outcome for SV insertions in our cohort. So far, the poor outcome in scalar dislocated CIs have been attributed to injuries in anatomical structures such as the Reissner’s membrane and the osseous spiral lamina [3]. Despite of possible damages to the scala media, the patients in our cohort profited from CI implantation. An explanation might be that the circumscribed trauma causes only punctual damage. In our cohort, each patient showed stable results or a gain in monosyllabic recognition on the implanted side, a decrease was not observed. There must be other causes for loss of residual hearing or unsuccessful rehabilitation. One cause may be the surgery itself or the surgical technique, as noted by Aschendorff et al. who observed surgical trauma to the fine structures of the cochlea, including fractures of the osseous spiral lamina and the modiolus during the cochleostomy [18]. Our cohort includes some cases with inner ear dysplasia and half of them achieved a benefit. One patient with cochlear dysplasia and electrode position in the SV achieved a postoperative outcome of 65% at 65 dB in quiet. Therefore, insertion into the SV seems to be an appropriate alternative to ST, especially in cases of ST obliteration, which supports the report by Kiefer et al. [7].

## Conclusion

The auditory outcome in our cohort does not support the assumption that ST insertion exclusively provides the best clinical outcome. Our results showed that SV insertion is an appropriate alternative in cases where ST insertion is complicated.

Identification of the fine and complex bony structures of the cochlea, such as the osseous spiral lamina to separate ST and SV, is possible in FDCT imaging due to its higher spatial resolution and reduced metal artifacts. Our results indicate that FDCT is a stable and reliable technique in CI imaging.

## Abbreviations

CI: Cochlear implant
FDCT: Flat-detector computed tomography
FMT: Freiburg monosyllabic test
IQR: Interquartile range
MSCT: Multi-slice computed tomography
MPR: Multiplanar reconstruction
R: Rater
SNHL: Sensorineural hearing loss
ST: Scala tympani
SV: Scala vestibuli
