# Correction to: Evaluation After Cochlear Implant Surgery

**DOI:** 10.1007/s00062-021-01074-6

**Published:** 2021-09-01

**Authors:** Annika Stock, Victoria Bozzato, Stephan P. Kloska, Alessandro Bozzato, Ulrich Hoppe, Joachim Hornung, Arnd Dörfler, Tobias Struffert

**Affiliations:** 1 Department of Neuroradiology, University Hospital Erlangen-Nuremberg, Erlangen, Germany; 2 Department of Neuroradiology, University Hospital Würzburg, Josef-Schneider-Straße 11, 97080 Würzburg, Germany; 3 Department of Otorhinolaryngology, Head and Neck Surgery, University Hospital Saarland Medical School, Homburg, Germany; 4 Department of Radiology, Hospital Fuerth, Fürth, Germany; 5 Department of Otorhinolaryngology, Head & Neck Surgery, University of Erlangen-Nuremberg, Erlangen, Germany; 6 Department of Neuroradiology, University Hospital Gießen, Gießen, Germany

**Correction to:**

**Clin Neuroradiol 2020**

10.1007/s00062-020-00922-1

The article “Evaluation After Cochlear Implant Surgery” written by Annika Stock, Victoria Bozzato, Stephan P. Kloska, Alessandro Bozzato, Ulrich Hoppe, Joachim Hornung, Arnd Dörfler and Tobias Struffert was originally published Online First without Open Access. After publication in volume 31, issue 2, page 367–372 the authors decided to opt for Open Choice and to make the article an Open Access publication. Therefore, the copyright of the article has been changed to © The Author(s) 2020 and the article is forthwith distributed under the terms of the Creative Commons Attribution 4.0 International License, which permits use, sharing, adaptation, distribution and reproduction in any medium or format, as long as you give appropriate credit to the original author(s) and the source, provide a link to the Creative Commons licence, and indicate if changes were made.

## Open Access

This article is licensed under a Creative Commons Attribution 4.0 International License, which permits use, sharing, adaptation, distribution and reproduction in any medium or format, as long as you give appropriate credit to the original author(s) and the source, provide a link to the Creative Commons licence, and indicate if changes were made. The images or other third party material in this article are included in the article’s Creative Commons licence, unless indicated otherwise in a credit line to the material. If material is not included in the article’s Creative Commons licence and your intended use is not permitted by statutory regulation or exceeds the permitted use, you will need to obtain permission directly from the copyright holder. To view a copy of this licence, visit http://creativecommons.org/licenses/by/4.0/.

